# Current status of sickle cell disease in India: how can you attenuate?

**DOI:** 10.1186/1755-8166-7-S1-I45

**Published:** 2014-01-21

**Authors:** Dipika Mohanty

**Affiliations:** 1 Apollo Hospitals, Bhubaneswar- 751 005, Odisha, India

## 

Sickle cell disease is the most prevalent monogenic disorder worldwide resulting from single DNA mutation in beta globin gene. The mapping on the pattern of its distribution in India has been studied to a great extent. However paucity of adequate data throughout the country regarding the clinical manifestations, natural history of the disorder and correlation with genotype and phenotype of the SCD cases is observed. This communication will focus on following aspects: (i) prevention and control of the SCD in India. (ii) the good clinical management with particular reference to pain during the vaso-occlussive crisis. (iii) neonatal screening and genetic counseling in SCD in tribals of India.

Between August 2009 and July 2010, 1668 newborns were enrolled and screened for SCD in Kalahandi district of Odisha. An average incidence of 17.62% of sickle cell trait (HbAS) was recorded for the area with the highest incidence observed in the tribal dominated part (19.03%). The data till date reveals a striking fact that more than 20 per thousand live births in the district are born with the disease. All the 34 cases of SCA detected were confirmed by parents testing, of which confirmation of 4 cases of compound heterozygosity for HbS and β-thalassaemia is an interesting finding.

For pain management with an informed approved consent in 15 patients treated at Apollo Hospital, Bhubaneswar we have given nitric oxide inhalation at 80ppm for 6-12hrs and 90% of patients responded well, pain score being relieved significantly >60%. In 2 patients there was recurrence of pain on 2^nd^ day and they were given again the same therapy. Another 1 patient did not respond very well to pain. On investigation she was found to have superficial vein thrombosis of left hand.

This NBS programme design provides scope for catering the benefit in rural areas and follow up for newborns. It also stresses on the acute need of such kind of extensive reach-out programme for early and confirmed detection of SCD in other places of the country. This will ultimately reduce the child mortality and morbidity in SCD. Nitric Oxide inhalation for pain relief in VOC of SCA is a very effective and less expensive method.

